# Investigating the *in-vitro* antimicrobial activities of sorghum [*Sorghum bicolor* (L.) Moench] phenolic extracts on liver abscess causing bacterial pathogens

**DOI:** 10.3389/fcimb.2025.1568504

**Published:** 2025-06-26

**Authors:** Harith M. Salih, Raghavendra G. Amachawadi, Qing Kang, Dmitriy Smolensky, Ramasamy Perumal, Sarah-Sexton Bowser, P. V. Vara Prasad, T. G. Nagaraja

**Affiliations:** ^1^ Department of Clinical Sciences, College of Veterinary Medicine, Kansas State University, Manhattan, KS, United States; ^2^ Department of Statistics, College of Arts and Sciences, Kansas State University, Manhattan, KS, United States; ^3^ Center for Grain and Animal Health Research, United States Department of Agriculture (USDA), Manhattan, KS, United States; ^4^ Kansas State University, Agriculture Research Center, Hays, KS, United States; ^5^ Department of Agronomy, Kansas State University, Manhattan, KS, United States; ^6^ Department of Diagnostic Medicine/Pathobiology, Kansas State University, Manhattan, KS, United States

**Keywords:** antibacterial activity, sorghum phenolic extracts, black sorghum, sumac sorghum, liver abscess, feedlot cattle

## Abstract

**Introduction:**

Liver abscesses that occur in finishing cattle fed high-grain, low-roughage diets, are of significant economic concern to the feedlot industry. The causative agents include both *Fusobacterium necrophorum* subspecies (*necrophorum* and *funduliforme*), *Trueperella pyogenes*, and *Salmonella enterica* serotype Lubbock. Tylosin, a macrolide antibiotic, is supplemented in the feed to reduce liver abscesses. Because of the concern with emergence of potential antimicrobial resistance, there is a need to find antibiotic alternatives. The aim of our study was to investigate the efficacy of phenolic compounds extracted from black and brown sumac sorghum extracts on liver abscess causing bacterial pathogens.

**Methods:**

Phenolic compounds were extracted by 75% aqueous acetone and total phenolic content was determined spectrophotometrically. Muller-Hinton broth (for *S. enterica* and *T. pyogenes*), and anaerobic Brain–Heart infusion broth (for *Fusobacterium*) with and without sorghum extracts (1 mg GAE/mL) were used. Growth was measured at 24 and 48 hours to determine bacterial concentration. Micro-broth dilution method was used to quantify growth inhibition.

**Results:**

Plant based phenolic compounds have the potential to be an antibiotic alternative to control liver abscesses. Sorghum [*Sorghum bicolor* (L.) Moench] grain phenolic compounds, have the potential to be one of these alternatives.

**Discussion:**

Our study demonstrated that the phenolic extracts of black and brown sumac sorghum exhibited antibacterial activities against the liver abscesses causing pathogens including both subspecies of *F. necrophorum* and *T. pyogenes* in a dose dependent manner, but not *S. enterica*. Sorghum phenolic compounds have the potential to be supplemented in the cattle feed to control liver abscesses.

## 1 Introduction

Liver abscesses (LA) are of significant economic concern to the feedlot industry. Liver abscesses accounts for approximately 67% of all liver abnormalities in cattle slaughtered in the United States. Severe abscesses can reduce carcass yield and quality, leading to a decrease in the value of the beef carcass by an average of $38 to $74 per animal. Losses related to liver condemnation alone are estimated to cost around $41.6 M annually in the United States (Brown and Lawrence, 2010; Herrick et al., 2022; McDaniel et al., 2023). The incidence of liver abscess in feedlot cattle in the United States range from 0 to 90%, but seem to vary greatly between different regions. It is generally ranging from 12 to 32% (Amachawadi and Nagaraja, 2016), and the average LA incidence is about 20.3% across all the US regions (Batista and Holland, 2022). *Fusobacterium necrophorum* is the primary causative agent, either alone or in association with other bacteria (Nagaraja and Chengappa, 1998). Two subspecies of *Fusobacterium*, called subsp. *necrophorum* and subsp. *funduliforme*, have been described, which differ in a number of characteristics, including ability to cause abscesses (Tadepalli et al., 2008). Other major bacterial species frequently isolated have included *Trueperella pyogenes* (Lechtenberg et al., 1988) and *Salmonella enterica* (Amachawadi and Nagaraja, 2015, 2022). Tylosin, supplemented in the feed, is the most commonly used antibiotic in the feedlot industry to prevent liver abscesses (FDA, 2022). Inclusion of tylosin in the feed reduces prevalence of liver abscesses (Weissend, 2018). Use of antibiotic in the feed raises concern of emergence and dissemination of gut bacteria that become resistant to tylosin. Therefore, a more effective intervention is warranted. Although tylosin is not used in human medicine, it belongs to the same class (macrolide) of antibiotic as erythromycin, which is widely used in human medicine. Macrolides belong to the macrolide-lincosamide-streptogramin B (MLSB) family, in which each antibiotic member has a slight difference from other members, which creates cross resistance (Leclercq and Courvalin, 2002; Roberts, 2008; Desmolaize et al., 2011). Because of FDA guidelines on the prudent use of antibiotics in livestock and with the implementation of Veterinary Feed Directive from January 1st, 2017, use of tylosin in the feed for prevention of liver abscesses has come under intense scrutiny. Therefore, research focus is needed to evaluate antibiotic alternatives for the control of liver abscesses in feedlot cattle.

Finishing cattle and lactating dairy cows in the United States are fed diets consisting of cereal grains and grain-based byproducts to meet energy de-mands and protein requirements. However, sorghum has unique attributes that could potentially be exploited to the economic advantage of cattle feeding operations. Sorghum is a drought tolerant and being grown mostly in dry lands it requires less water and agronomical inputs than corn. Sorghum contains high value phenolic compounds with bioactive properties (Dykes et al., 2005; Gu et al., 2007; Hagerman et al., 1988; Proestos et al., 2005) in the bran that have the potential for improving animal productivity and health. Sorghum was selected for investigation due to its high content of polyphenolic compounds, especially in pigmented varieties such as black and sumac sorghum. These polyphenols possess well-documented antimicrobial, antioxidant, and anti-inflammatory activities, which are potentially beneficial in reducing the microbial burden associated with liver abscess formation. Moreover, sorghum is an economically important, drought-tolerant cereal crop that is widely available in many cattle-feeding regions, making it a practical and scalable dietary intervention. Sorghum grain is also heavily utilized in ethanol production. The extraction of sorghum phenolic compounds from sorghum bran could be incorporated into an overall ethanol processing scheme for the development of high value sorghum. In recent years, increasing attention has been directed toward the polyphenol content of sorghum due to its potential health-promoting properties and role in disease prevention. Polyphenols, recognized for their potent antioxidant activity, can scavenge free radicals and modulate signaling pathways implicated in various pathological processes (Pontieri et al., 2021). However, the levels of these bioactive compounds vary significantly among different sorghum brans. Notably, black and sumac sorghum brans contain higher concentrations of polyphenols and antioxidants, whereas white and mycogen sorghum brans as well as commonly consumed cereal brans such as wheat, oat, and rice exhibit comparatively lower levels (Burdette et al., 2010).

Earlier studies have reported that sorghum phenolic compounds, including tannins, have antimicrobial effects against certain bacteria, including *Staphylococcus aureus*, *Salmonella*, *Escherichia coli*, *Klebsiella pneumoniae*, and yeasts (Sulieman et al., 2007; Kil et al., 2009). Also, *in-vitro* studies have shown that sorghum phenolic compounds have antioxidant (Herald et al., 2012), anti-inflammatory (Burdette et al., 2010) and anticancer (Alara et al., 2021) activities. The bioactive properties of sorghum bran phenolic compounds may have useful applications in cattle productions systems, including beneficial impact on ruminal fermentations applicable to feedlot cattle and lactating dairy cows for the prevention of liver abscesses. The hypothesis is that the available specialty grain sorghums, such as black and brown sumac, are genetically rich in phenolic compounds and could aid in controlling bacterial pathogens responsible for liver abscesses. The main objectives of this study were to evaluate and quantify antimicrobial activities of specialty grain sorghum phenolic extracts against bacterial pathogens responsible for liver abscesses.

## 2 Materials and methods

### 2.1 Plant extracts

Specialty sorghum bran from black (NLM-200304-33) and brown sumac (NLM-200227-SB) milled products from NuLife company (NuLife Market LLC., Scott City, KS) were used in this study.

### 2.2 Study design

A randomized complete block design with repeated measurements for NLM-200304–33 and NLM-200227-SB at a given concentration for each bacterial species was used. The treatment groups were in a 2-way factorial arrangement, where bacterial strain and growing condition were factors. The replication of a complete set of treatment groups formed a block. Bacterial concentrations were measured in OD (optical density) and log10(CFU/mL).

### 2.3 Extraction of sorghum phenolic extracts

The extraction was carried out as per the previously published procedure using a solid-liquid extraction method (Alara et al., 2021). Briefly, the brans from both black and brown sumac sorghum were ground before extraction. Approximately, 100 g of each bran was mixed with 500 mL of 75% Acetone (Fisher Scientific, Fair Lawn, NJ). The mixture was continuously stirred for 2 h, stored for 24 h at -20°C, and then centrifuged at 3345 G for 10 minutes using a refrigerated centrifuge. The supernatant was then subjected for solvent (acetone) removal process by using the rotary evaporator at 40°C (Toolots^®^, Colton, CA). The suspension was freeze dried, and the product sampled to measure the final total phenolic content (TPC) concentration before storing the freeze-dried powder at -20°C until further use (Tian and Li, 2018).

### 2.4 Measurement of total phenolic contents

After the extraction process, 0.1 mL of resultant supernatant was mixed thoroughly with 7.9 mL of deionized water and 0.5 mL of Folin-Ciocalteu reagent. The mixture was allowed to settle at room temperature for 5–10 minutes before adding 1.5 mL of Na_2_CO_3_ solution (20% w/v). After adding Na_2_CO_3_ solution, the mixture was kept at room temperature for 2 h and record the absorbance of the mixture at 765 nm using the VWRUV1600-PC Spectrophotometer (Randor, PA). The Gallic acid was used as an external standard for spectrophotometer results and expressed as microgram gallic acid equivalence (GAE) per gram of the original supernatant sample (mg GAE/g) (Tian and Li, 2018).

### 2.5 Bacterial strains

The bacteria tested included five strains each of *F. necrophorum* subsp. *necrophorum* (2016-13#55, 2016-13#69, 2018-13#23, 2018-13#24, and 2018-13#37) *F. necrophorum* subspecies *funduliforme* (2018-1#6, 2018-1#7, 2018-13#2, 2018-13#16, and 2018-13#35), *S. enterica* serotype Lubbock (2016-13#26, 2016-13#33, 2016-13#34, 2016-13#36, and 2016-13#38), and *T. pyogenes* (2016-13#306, 2016-13#311, 2016-13#315, 2016-13#318, and 2016-13#319). These strains were all isolated from liver abscesses in feedlot cattle.

### 2.6 Antimicrobial susceptibility testing of black and sumac sorghum extracts

#### 2.6.1 Preparation of bacterial inocula

All the bacterial species were grown on blood agar plates (Remel, Lenexa, KS). *Fusobacterium* subspecies were incubated anaerobically at 39°C for 48 h, while the *Salmonella* incubated aerobically at 37°C for 24 h, and the *T. pyogenes* incubated under the CO_2_ condition at 37°C for 48 h. Colonies were suspended into the PRAS (prereduced anaerobically sterilized)-BHI (Brain Heart Infusion) broth (Becton Dickinson, Sparks, MD) for *Fusobacterium* and MH (Mueller-Hinton) broth for both *Salmonella* and *Trueperella*. The inoculum concentration was adjusted to 0.5 McFarland Standards (Remel, Lenexa, KS). The susceptibility testing was according to the Clinical and Laboratory Standard Institute (CLSI) guidelines (CLSI, 2023).

#### 2.6.2 Broth macro-dilution method

The antimicrobial activities of the sorghum phenolic extracts were determined by macro-dilution method (Febler et al., 2018). Unless otherwise mentioned, PRAS-BHI and MH broth were used for both *Fusobacterium* subspecies and *S.* Lubbock and *T. pyogenes*, respectively. Bacteria with and without sorghum extracts were grown and serially diluted (10-fold) at 24 and 48 h post incubation. The bacterial growth was measured at 24 and 48 h at 600 nm followed by spread-plating for bacterial enumeration. The procedure was repeated with different bacterial inoculum. Dimethylsulfoxide (DMSO) served as the negative or a solvent control.

#### 2.6.3 Broth micro-dilution method

The minimum inhibitory concentration (MIC) of sorghum phenolic extracts, chlortetracycline (Sigma Aldrich, St. Louis, MO) and tylosin (Sigma Aldrich) was determined by microbroth dilution method following the CLSI guidelines (CLSI, 2023). Both chlortetracycline and tylosin antibiotics served as antibiotic controls (positive control) for comparison with the sorghum phenolic extracts. Antibiotic stock solutions were prepared as per the manufacturer’s guidelines to achieve a concentration of 1,000 µg/mL, based on their respective potencies. The sorghum phenolic extracts and antibiotics were tested at a concentration of 100, 50, 25, 12.5, 6.25, 3.125, 1.56, 0.78, 0.39, and 0.195 µg/mL. The assay was conducted in 96-well micro titer plates (Becton and Dickinson, Franklin Lakes, NJ). Plates were incubated under anaerobic (*Fusobacterium*), CO_2_ (*Trueperella*), and aerobic (*Salmonella*) conditions. Dimethylsulfoxide (DMSO) served as the negative or a solvent control. Results were recorded based on bacterial growth or no growth. The procedure was repeated with different bacterial inoculum.

#### 2.6.4 Statistical analysis

Bacterial growth was subjected to the linear mixed model analysis. OD change from control refers to the absolute difference between a bacterial strain and the control in the same growing condition, time and replication. Fixed effects of the model included replication (Rep 1, Rep 2), strain (varying among experiments), growing condition (medium + bacteria, medium + DMSO + bacteria, medium + sorghum phenolic compound + bacteria), time (24 and 48 h, and when applicable, 0 and 12 h) and all the 2-way and 3-way interactions between strain, growing condition and time. The random effect was the combination of replication, strain and growing condition. Variance-covariance of the random effect was taken as compound symmetry heterogeneous with respect to growing condition. When the 3-way interaction was not significant, the effect of growing condition was evaluated at a given time; otherwise, the effect of growing condition was evaluated at a given time and for a given strain. The least-square means (LSMs) and standard errors (SEMs) were used to assess the fixed effects. All tests were two-sided and were conducted at the 0.05 level. No multiplicity adjustment was applied. Statistical analyses were performed using SAS (version 9.4; Cary, NC software) MIXED procedure with the DDFM=KR option in the MODEL statement.

## 3 Results

### 3.1 Total phenolic content

The total phenolic contents of black and brown sumac sorghum were 258.3 and 374.9 mg gallic acid equivalents (GAE)/gram, respectively.

### 3.2 Broth macro-dilution method

Both, black and brown sumac sorghum phenolic extracts were tested at 0.1 mg GAE/mL and 1 mg GAE/mL concentrations. At 0.1 mg GAE/mL concentration, showed a varying degree of inhibition against these bacterial strains ranging from complete inhibition of *T. pyogenes* (P < 0.0001), partial inhibition of subsp. *necrophorum* (P < 0.0001) and no effect on subsp. *funduliforme* and *S.* Lubbock (P > 0.05). At 1 mg GAE/mL concentration both phenolic extracts inhibited the growth of both subspecies of *F. necrophorum* (*necrophorum* and *funduliforme*), and *T. pyogenes* (P < 0.0001), but not *S.* Lubbock (P > 0.05). The effect of black and brown sumac sorghum phenolic extracts at 0.1 and 1 mg GAE/mL concentration on the bacterial growth showed significant interaction effect of growth condition and time (P < 0.0001) for all the tested bacterial species. However, 3-way interaction (bacteria, growth condition and time) was significant for brown sumac sorghum against subsp. *funduliforme* at both 0.1 mg GAE/mL (P = 0.0347) and 1 mg GAE/mL (P = 0.0017) concentrations (**Table 1**).

**Table 1:** Effect of black and brown sumac sorghum phenolic extracts on the growth (optical density at 600 nm) of liver abscess causing pathogens.

Bacteria	Sorghum	Concentration (mg/ml)	OD (600 nm)				Rep	Str	GC	Str*GC	T	Str*T	GC*T	Str*GC*T
			0 h	12 h	24 h	48 h								
*F. necrophorum*	Black	0.1	0.482	0.718	0.596	0.708	0.0105	0.6708	<0.0001	0.3535	<0.0001	0.9992	<0.0001	1.0000
*F. funduliforme*	Black	0.1	–	0.646	0.712	0.748	0.1777	0.0459	<0.0001	0.2028	<0.0001	0.5989	<0.0001	0.3992
*T. pyogenes*	Black	0.1	0.368	0.436	0.398	0.460	–	–	–	–	–	–	–	–
*S.* Lubbock	Black	0.1	–	–	–	–	–	–	–	–	–	–	–	–
*F. necrophorum*	Black	1	2.354	2.370	2.372	2.368	–	–	–	–	–	–	–	–
*F. funduliforme*	Black	1	3.00	3.00	3.00	3.00	–	–	–	–	–	–	–	–
*T. pyogenes*	Black	1	2.090	2.220	2.222	2.234	–	–	–	–	–	–	–	–
*S.* Lubbock	Black	1	2.420	2.418	2.104	2.086	–	–	–	–	–	–	–	–
*F. necrophorum*	Brown	0.1	0.25	0.336	0.502	0.466	0.0051	0.7429	0.0003	0.9980	<0.0001	0.9922	<0.0001	0.9906
*F. funduliforme*	Brown	0.1	0.26	0.256	0.268	0.266	0.1928	0.7893	<0.0001	0.1861	<0.0001	0.0153	<0.0001	0.0347
*T. pyogenes*	Brown	0.1	0.142	0.160	0.156	0.178	–	–	–	–	–	–	–	–
*S.* Lubbock	Brown	0.1	–	–	–	–	–	–	–	–	–	–	–	–
*F. necrophorum*	Brown	1	1.514	1.520	1.522	1.528	–	–	–	–	–	–	–	–
*F. funduliforme*	Brown	1	2.116	0.224	0.574	1.242	<0.0001	0.0016	<0.0001	0.0008	<0.0001	0.0262	<0.0001	0.0017
*T. pyogenes*	Brown	1	0.762	0.882	0.988	1.124	–	–	–	–	–	–	–	–
*S.* Lubbock	Brown	1	0.658	1.15	1.39	1.744	<0.0001	0.9428	<0.0001	0.4721	<0.0001	0.8560	<0.0001	0.8945

Rep, Replication; Str, Strain; GC, Growth condition; T, Time.

*Growth condition = Bacteriological culture medium alone or in combination bacteria; Bacteriological culture medium alone or in combination with DMSO (Dimethyl sulfoxide); Bacteriological culture medium alone or in combination with sorghum phenolic compounds.

The effect of black and brown sumac sorghum phenolic extracts at 1 mg GAE/mL concentration on the bacterial growth showed significant effect of growth condition (P < 0.0001) for all the tested bacterial species. We did not find 2-way and or 3-way interaction for bacterial counts (**Table 2**).

**Table 2:** Effect of black and brown sumac sorghum phenolic extracts on the growth (colony forming units; CFU) of liver abscess causing pathogens.

Bacteria	Sorghum	mg/ml	CFU/ml		Rep	Str	GC	Str*GC	T	Str*T	GC*T	Str*GC*T
			24 h	48 h								
*F. necrophorum*	Black	0.1	**-**	**-**	**-**	**-**	**-**	**-**	**-**	**-**	**-**	**-**
*F. funduliforme*	Black	0.1	**-**	**-**	**-**	**-**	**-**	**-**	**-**	**-**	**-**	**-**
*T. pyogenes*	Black	0.1	**-**	**-**	**-**	**-**	**-**	**-**	**-**	**-**	**-**	**-**
*S.* Lubbock	Black	0.1	**-**	**-**	**-**	**-**	**-**	**-**	**-**	**-**	**-**	**-**
*F. necrophorum*	Black	1	8.17x10^6^	4.49x10^6^	<0.0001	0.4682	0.0009	0.8308	0.2168	0.5000	0.9994	0.6712
*F. funduliforme*	Black	1	1.07x10^8^	5.32x10^7^	0.6434	0.8589	0.0197	0.6296	0.3861	0.6261	0.8439	0.7250
*T. pyogenes*	Black	1	–	–	–	–	–	–	–	–	–	–
*S.* Lubbock	Black	1	9.58x10^7^	4.40x10^7^	0.6950	0.9036	<0.0001	0.9335	0.0509	0.5654	0.4895	0.5909
*F. necrophorum*	Brown	0.1	**-**	**-**	–	–	–	–	–	–	–	–
*F. funduliforme*	Brown	0.1	**-**	**-**	–	–	–	–	–	–	–	–
*T. pyogenes*	Brown	0.1	**-**	**-**	–	–	–	–	–	–	–	–
*S.* Lubbock	Brown	0.1	**-**	**-**	–	–	–	–	–	–	–	–
*F. necrophorum*	Brown	1	**-**	**-**	–	–	–	–	–	–	–	–
*F. funduliforme*	Brown	1	1.08x10^8^	6.17x10^7^	0.0178	0.9735	0.0275	0.6399	0.6866	0.7680	0.4627	0.6748
*T. pyogenes*	Brown	1	–	–	–	–	–	–	–	–	–	–
*S.* Lubbock	Brown	1	2.28x10^8^	1.29x10^8^	0.3304	0.1711	<0.0001	0.0594	0.0516	0.5520	0.3589	0.3053

Rep, Replication; Str, Strain; GC,; Growth condition; T, Time.

**Figure 1:**
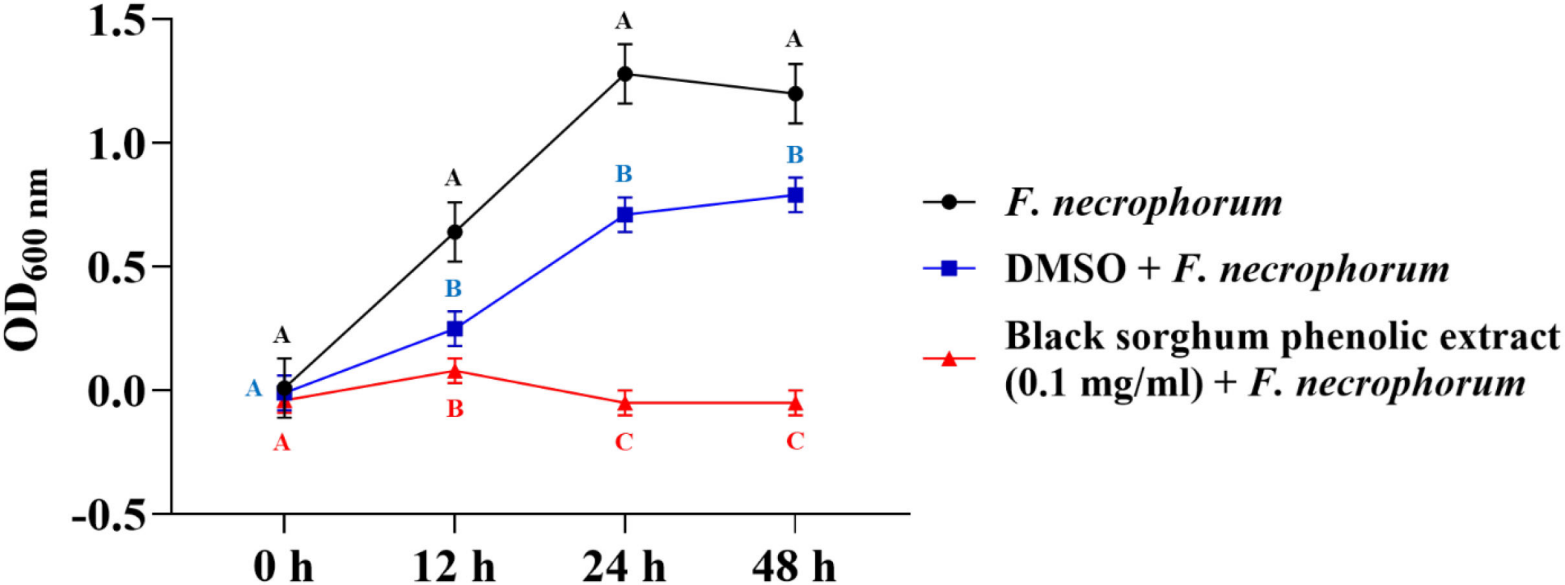
Growth rate (OD600 nm) of *Fusobacterium necrophorum* ssp. *necrophorum* in the presence of black sorghum phenolic extract (0.1 mg/ml). Different letters indicate statistically significant differences (P < 0.05).

**Figure 2:**
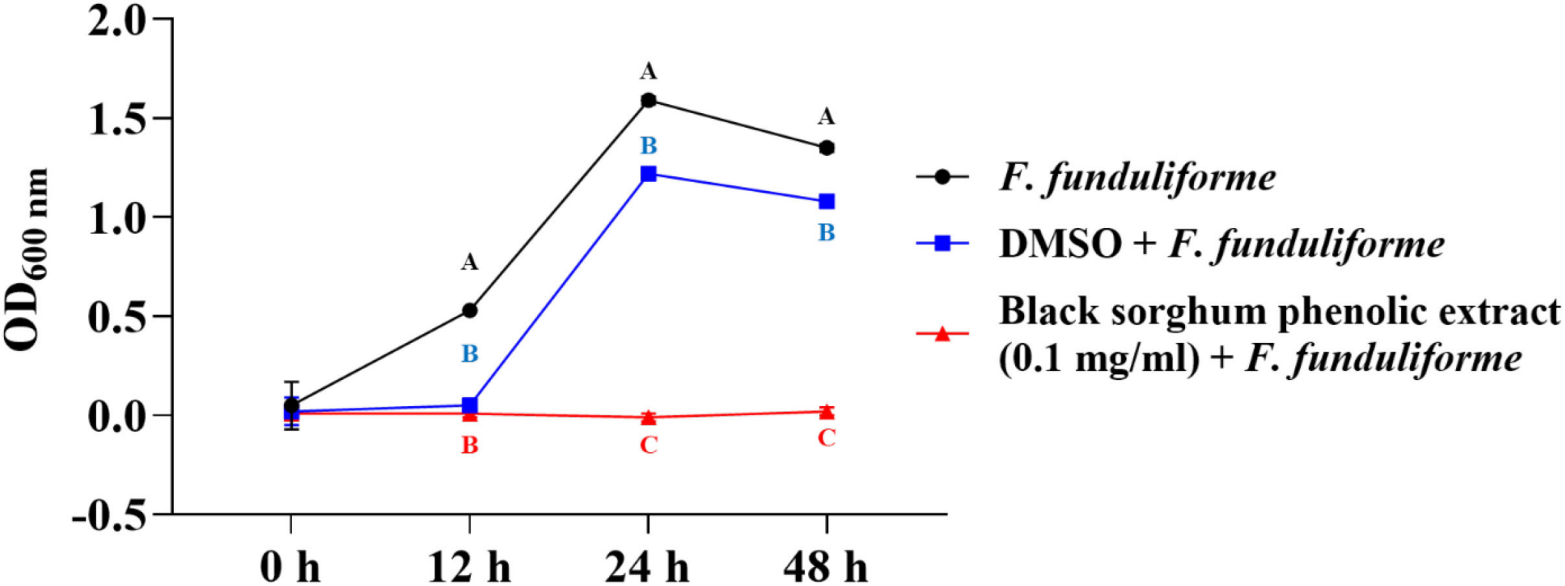
Growth rate (OD600 nm) of *Fusobacterium necrophorum* ssp. *funduliforme* in the presence of black sorghum phenolic extract (0.1 mg/ml). Different letters indicate statistically significant differences (P < 0.05).

**Figure 3:**
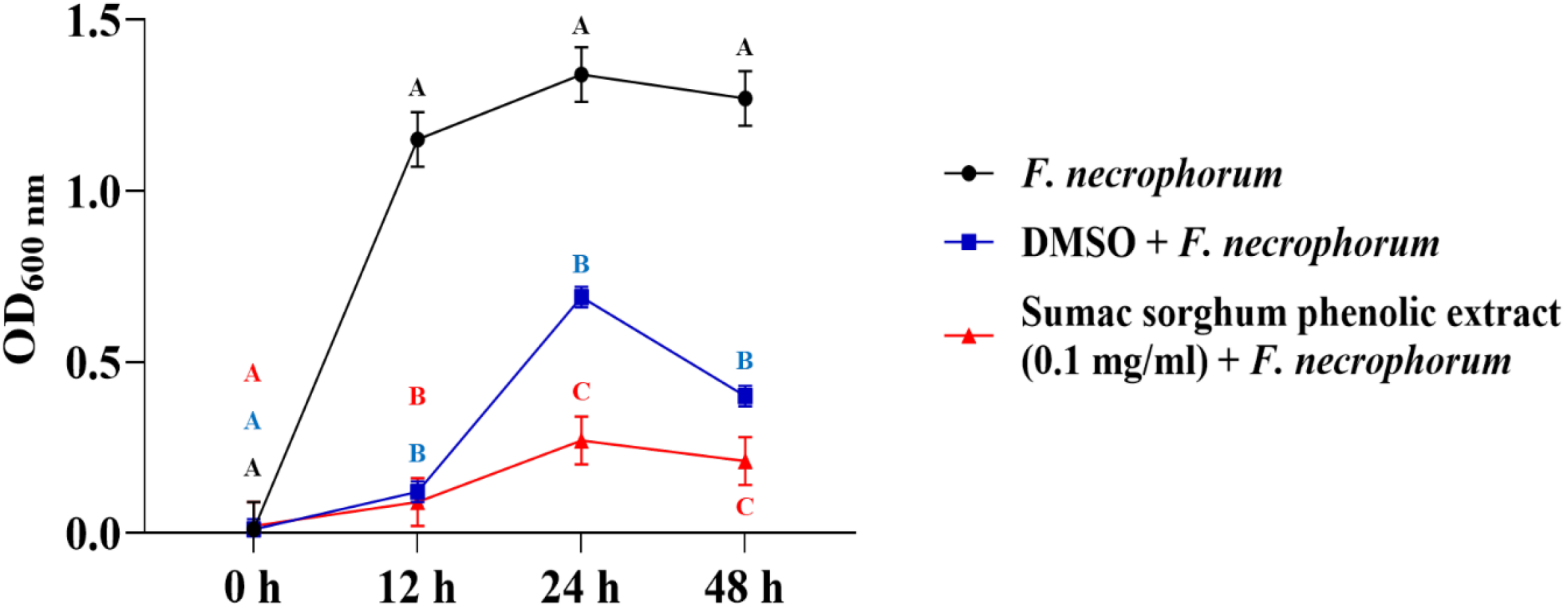
Growth rate (OD600 nm) of *Fusobacterium necrophorum* ssp. *necrophorum* in the presence of brown sumac sorghum phenolic extract (0.1 mg/ml). Different letters indicate statistically significant differences (P < 0.05).

**Figure 4:**
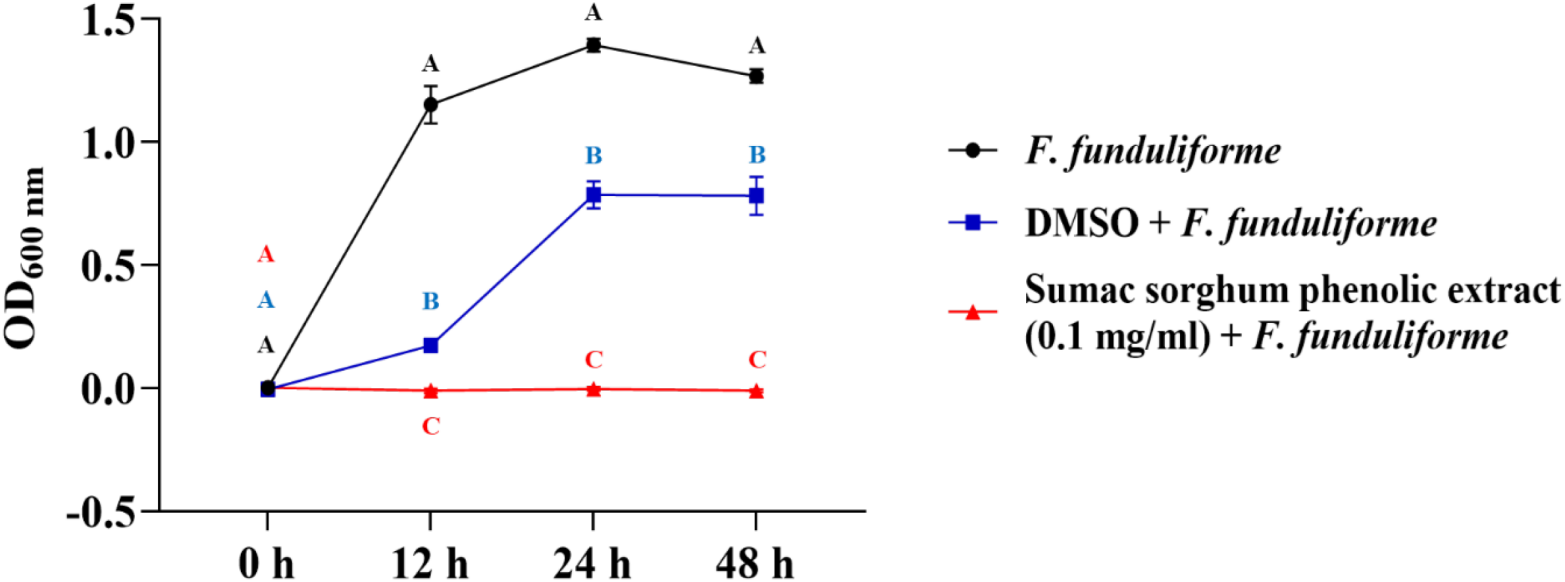
Growth rate (OD600 nm) of *Fusobacterium necrophorum* ssp. *funduliforme* in the presence of brown sumac sorghum phenolic extract (0.1 mg/ml). Different letters indicate statistically significant differences (P < 0.05).

**Figure 5:**
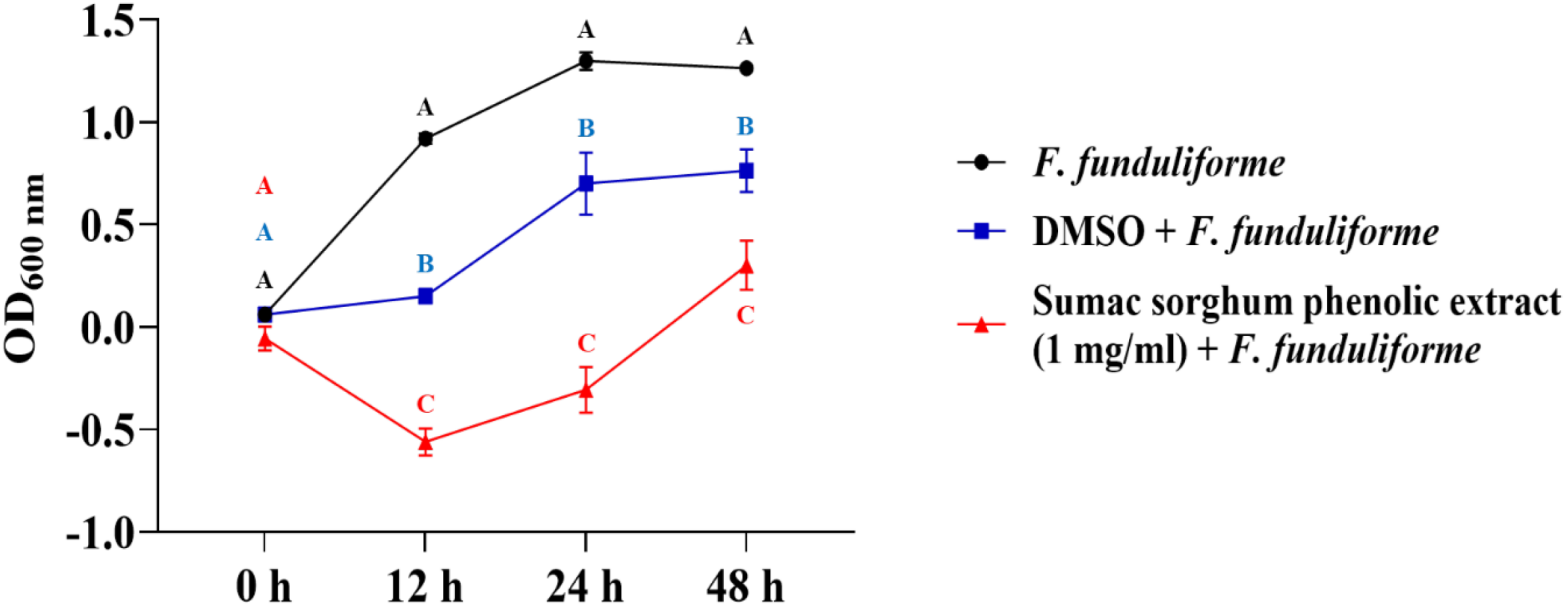
Growth rate (OD600 nm) of *Fusobacterium necrophorum* ssp. *funduliforme* in the presence of brown sumac sorghum phenolic extract (1 mg/ml). Different letters indicate statistically significant differences (P < 0.05).

**Figure 6:**
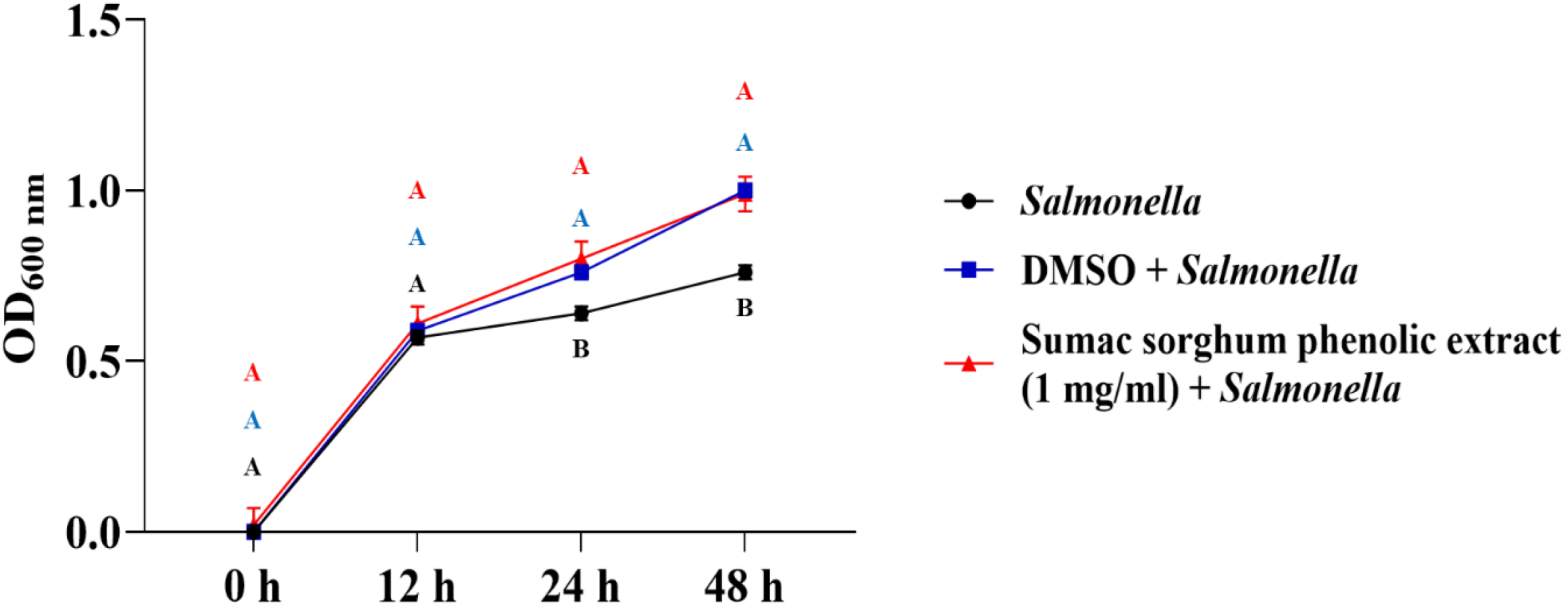
Growth rate (OD600 nm) of *Salmonella enterica Lubbock* in the presence of brown sumac sorghum phenolic extract (1 mg/ml). Different letters indicate statistically significant differences (P < 0.05).

The bacterial growth was also monitored by measuring the optical density (OD) difference from the control, with different growth conditions: bacterial control, dimethyl sulfoxide (DMSO) + bacteria, and phenolic extract + bacteria. The least square means (± SE) of growth, measured as absorbance, for the black and brown sumac sorghum phenolic extract in the concentration of 0.1 mg GAE/mL and 1 mg GAE/mL at 0, 12, 24, and 48 hours of incubations are shown in (**Figures 1**–**6**). The results demonstrate that phenolic extracts from black and brown sorghum significantly inhibited the growth of *F. necrophorum* subsp. *necrophorum* and *funduliforme* at 24 and 48 hours, with the most notable reductions observed with black sorghum extract. At 24 hours, the OD difference for *F. necrophorum* subsp. *necrophorum* treated with black sorghum extract was -0.05, and it remained the same at 48 hours, indicating sustained inhibition. In contrast, the extracts had little to no inhibitory effect on *S. enterica* serotype Lubbock, suggesting that sorghum phenolic extracts are more effective against *Fusobacterium* species.

**Figure 7:**
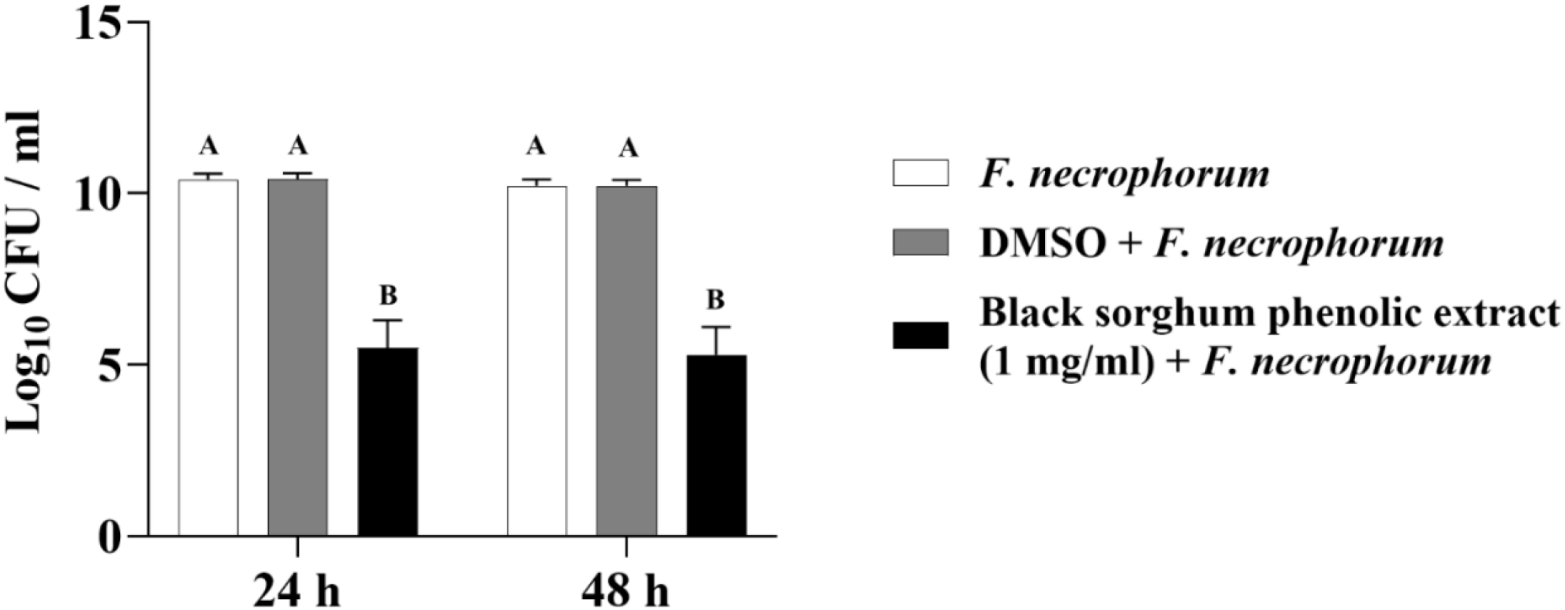
Effect of black sorghum phenolic extract (1 mg/ml) on the growth rate (log10 CFU/ml) of *Fusobacterium necrophorum* ssp. *necrophorum*. Different letters represent statistically significant differences (P < 0.05).

**Figure 8:**
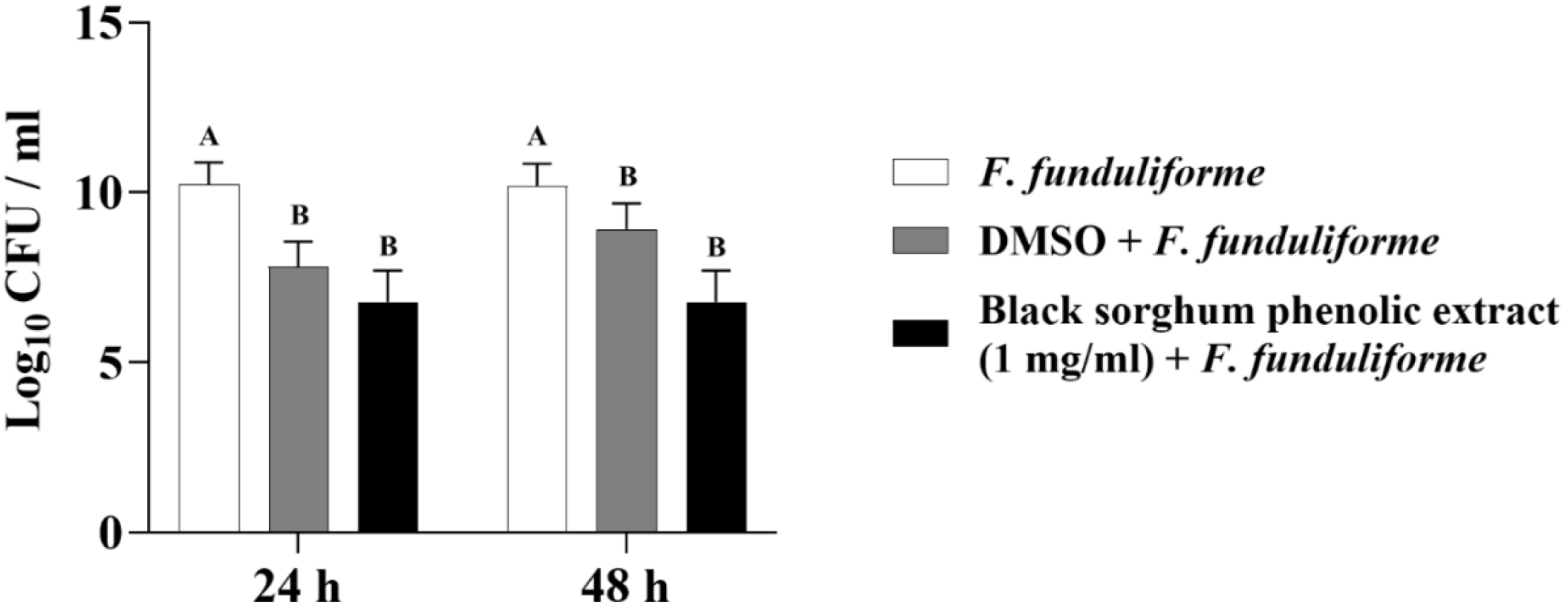
Effect of black sorghum phenolic extract (1 mg/ml) on the growth rate (log10 CFU/ml) of *Fusobacterium necrophorum* ssp. *funduliforme*. Different letters represent statistically significant differences (P < 0.05).

**Figure 9:**
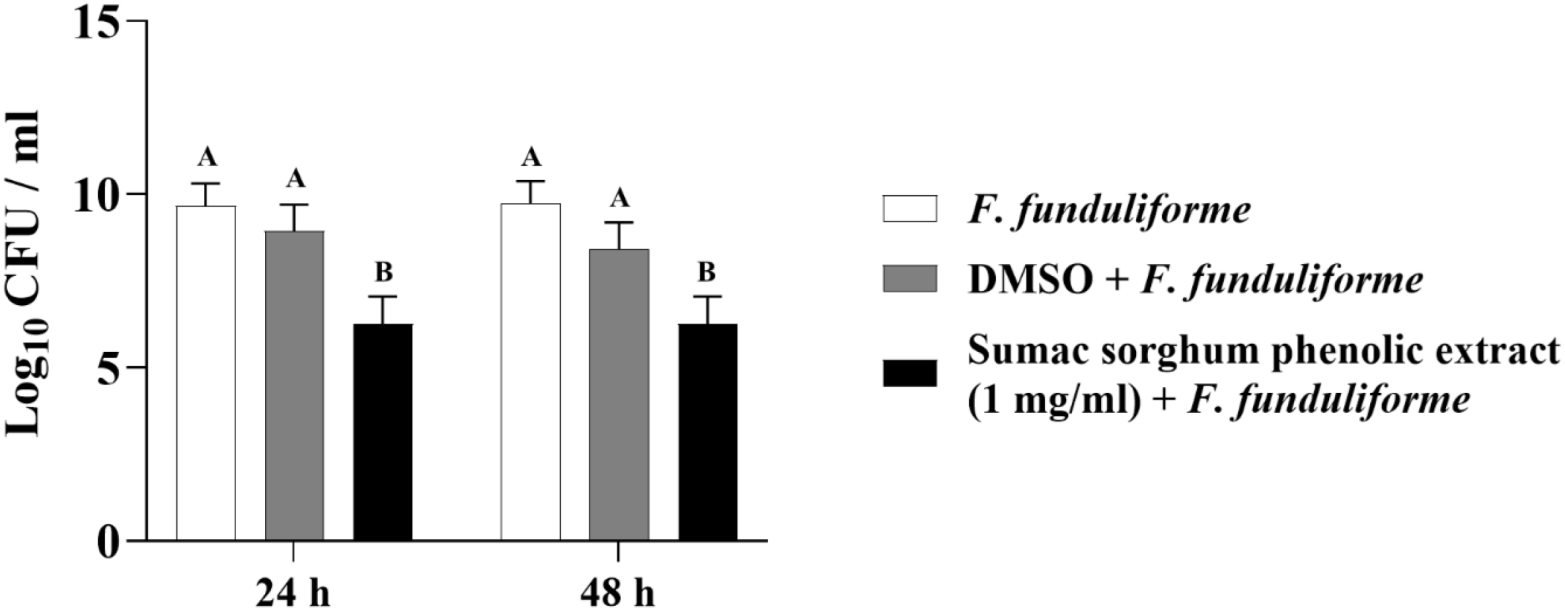
Effect of brown sumac sorghum phenolic extract (1 mg/ml) on the growth rate (log10 CFU/ml) of *Fusobacterium necrophorum* ssp. *funduliforme*. Different letters represent statistically significant differences (P < 0.05).

**Figure 10:**
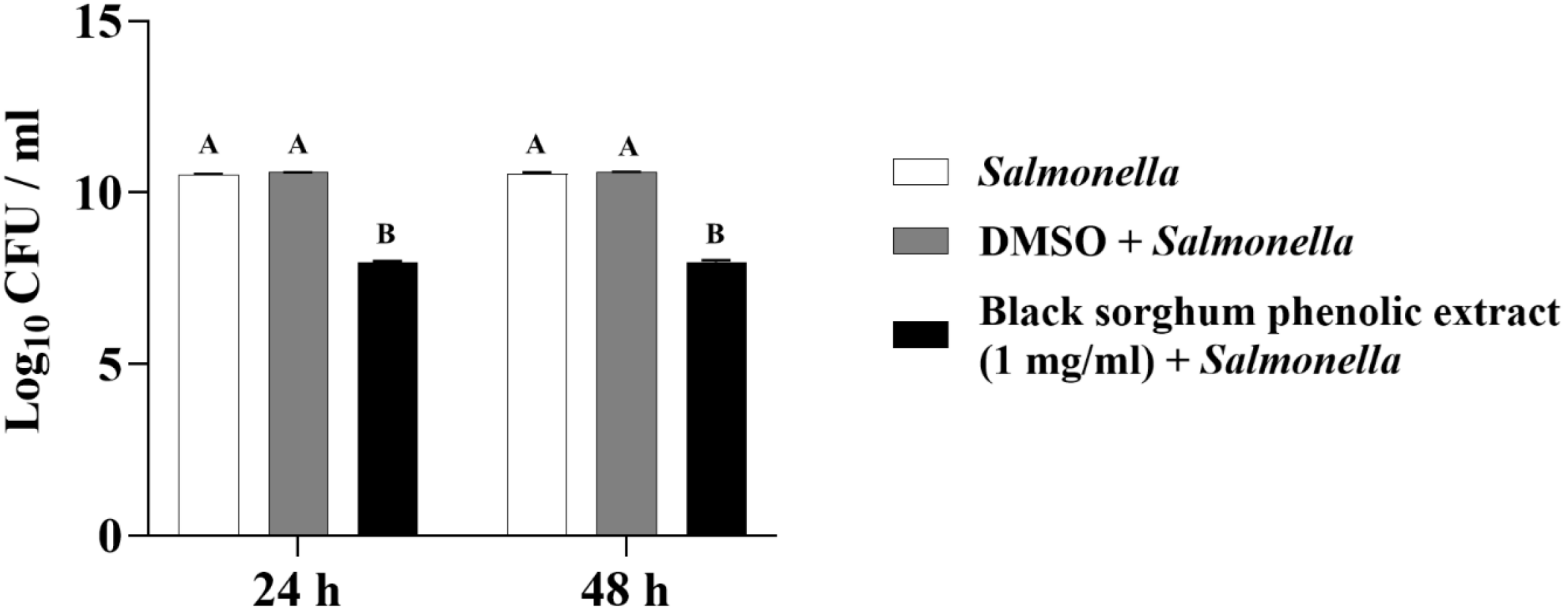
Effect of black sorghum phenolic extract (1 mg/ml) on the growth rate (log10 CFU/ml) of *Salmonella enterica Lubbock*. Different letters represent statistically significant differences (P < 0.05).

**Figure 11:**
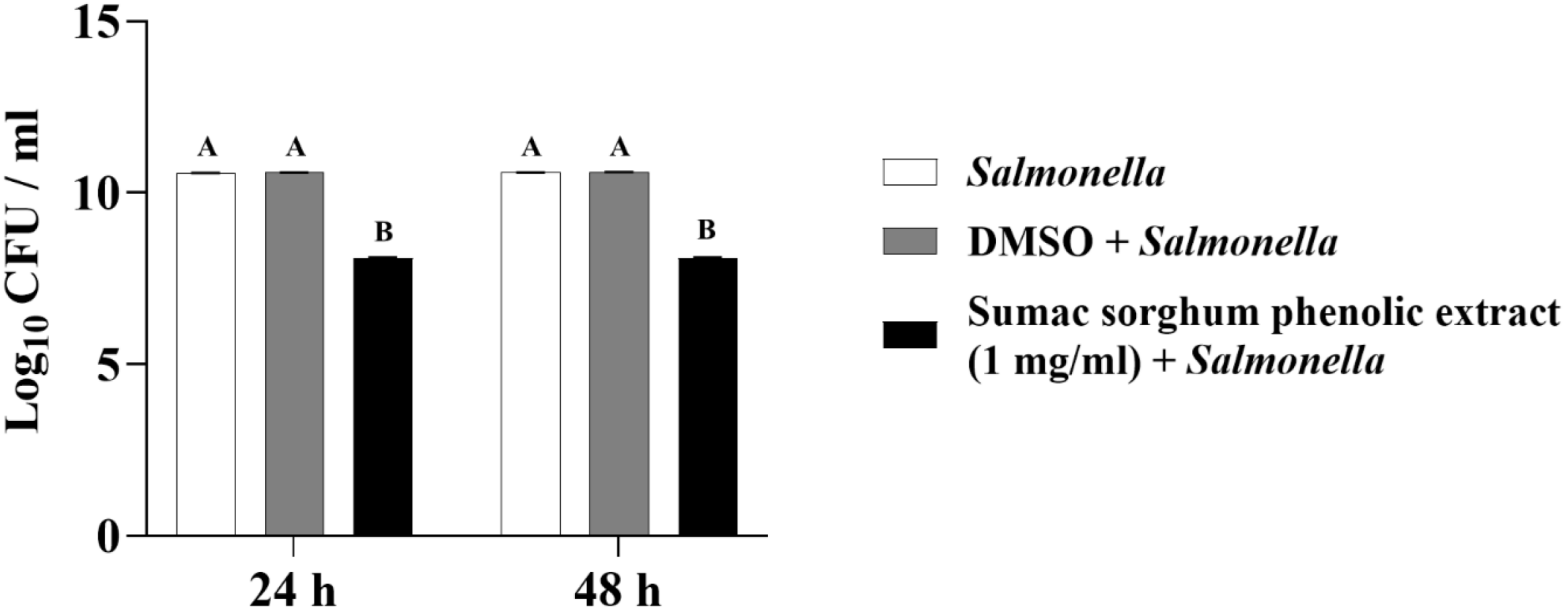
Effect of brown sumac sorghum phenolic extract (1 mg/ml) on the growth rate (log10 CFU/ml) of *Salmonella enterica Lubbock*. Different letters represent statistically significant differences (P < 0.05).

The least square means (± SE) of bacterial counts (CFU/mL) at the concentration of 1 mg GAE/mL at 24, and 48 hours of incubation are shown in (**Figures 7**–**11**). The results show that both black and brown sorghum phenolic extracts significantly inhibited the growth of liver abscess-causing pathogens, including *F. necrophorum* and *S. enterica* serotype Lubbock, at 24 and 48 hours. In all cases, the phenolic extracts reduced bacterial counts compared to the controls and DMSO-treated groups (P < 0.05), demonstrating their potential antibacterial properties. These results indicate that both black and brown sorghum phenolic extracts have potent inhibitory effects on the growth of liver abscess-causing pathogens, with statistically significant reductions in CFU compared to the controls across the 24- and 48-hour incubation periods. Black sorghum exhibited slightly stronger effects overall, particularly against *F. necrophorum* subsp. *necrophorum*, where bacterial growth was notably reduced at both time points.

### 3.3 Broth micro-dilution method

**Figure 12:**
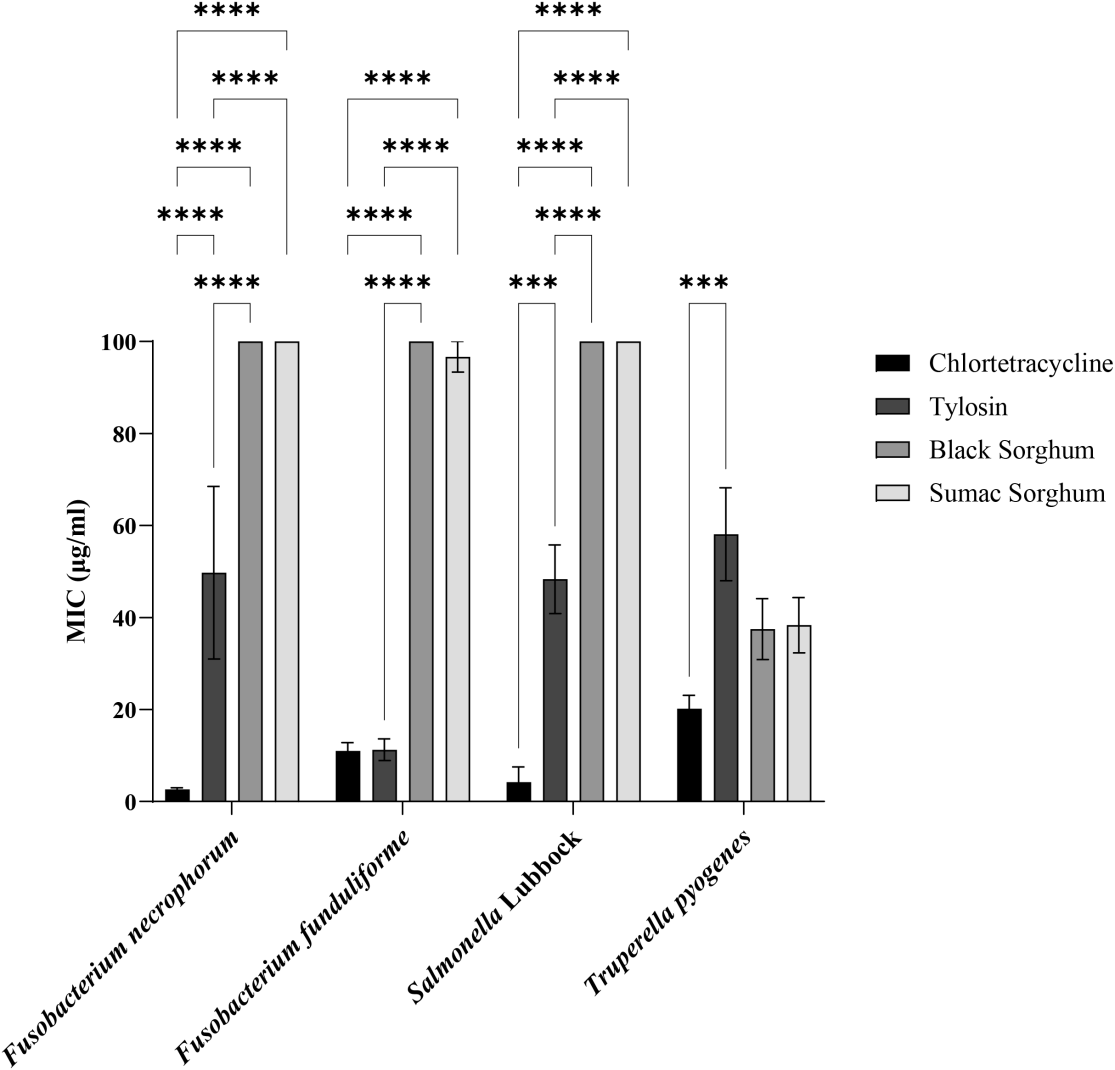
Minimum Inhibitory Concentrations (MIC; µg/mL) of black sorghum, brown sumac sorghum, tylosin and chlortetracycline against liver abscess causing bacterial pathogens. MIC values are presented as mean ± SEM, with statistical significance denoted as ***P<0.001 and ****P<0.0001.

Subsp. *necrophorum* and *funduliforme*, and *Salmonella* Lubbock strains were resistant to black and brown sumac sorghum phenolic compounds (≥100 µg/mL) at 1 mg GAE/mL concentrations. The subsp. *necrophorum* strains had an MIC value of 23.6 and 49.7 µg/mL for chlortetracycline and tylosin, respectively. The subsp. *funduliforme* strains had an MIC value of 11 and 11.2 µg/mL for chlortetracycline and tylosin, respectively. The *T. pyogenes* strains had an MIC value of 42.5 µg/mL and 23.7 µg/mL for black and brown sumac sorghum phenolic compounds, respectively. The *T. pyogenes* strains had an MIC value of 20 and 57.9 µg/mL for chlortetracycline and tylosin, respectively. The *Salmonella* isolates had an MIC value of 4.2 and 49.8 µg/mL for chlortetracycline and tylosin, respectively. The bar graph displaying the MIC values along with the standard error of the mean (SEM) for all bacteria is presented in **Figure 12**.

## 4 Discussion

Liver abscesses in feedlot cattle are commonly linked to high dietary concentrations of cereal grains, which can lead to lower ruminal pH and subsequent inflammation and damage to the ruminal epithelial tissue. This damage facilitates the entry of bacteria into the portal blood, resulting in abscess formation in the liver (Amachawadi and Nagaraja, 2022). While antibiotics like tylosin are effective in controlling abscesses, concerns about antibiotic use in livestock are driving interest in alternative methods. The widespread use of antibiotics has led to the development and spread of antibiotic resistance, posing a significant public health challenge. Therefore, even with efforts to reduce antibiotic use, it is essential to find alternative solutions that replicate the benefits of antibiotics to ensure global food security. Given the concerns surrounding the loss of medically important antibiotics due to regulatory restrictions and the rise of antimicrobial resistance (AMR), there is an urgent need to develop innovative strategies and tools that enhance animal health and improve production efficiency (Callaway et al., 2021). Due to the lengthy and expensive process of discovering new antibiotics, researchers worldwide have focused on exploring the antimicrobial properties of various natural sources. Investigations in this area have shown that phytochemicals extracted from natural plant sources can be used in combination with antibiotics, enhancing their effectiveness and allowing for reduced dosages (Barbieria et al., 2017).

Sorghum, commonly used as a feed grain in the United States and recognized as the fifth leading cereal crop globally, has garnered attention for its potential health and pharmacological benefits. These benefits include lowered cholesterol, reduced cardiovascular disease risk, antioxidant, ant inflammatory, and anticarcinogenic properties. Specialty sorghum varieties, particularly those with red, brown, or black pericarp, contain high levels of phenolic compounds such as phenolic acids, flavonoids, and condensed tannins, which contribute to their health-promoting effects (Xiong et al., 2019; Xiong et al., 2022). These phenolic compounds act as antioxidants, protecting the plant against insects and diseases, and play a significant role in reducing the risk of chronic conditions such as inflammatory bowel disease, atherosclerosis, cancer, and rheumatoid arthritis by neutralizing free radicals. Brown and black specialty grain sorghum have higher antioxidant activities than tan/purple types. Specialty sorghum grains with a thick pericarp layer have higher anti-oxidant compounds than those with a thin pericarp (Dykes et al., 2005). This pericarp layer with red, black, white or brown color is either non-pigmented or pigmented and it depends on the presence of tannins. Sorghum polyphenolic compounds have been characterized and quantified in five sorghum genotypes by high performance liquid chromatography (Punia et al., 2020). The results showed that red and brown pericarp genotypes had higher proanthocyanidin and phenolic contents, which result in high antioxidant activities. The specific phenolic acids with higher antioxidant activity in brown and red genotypes include taxifolin, apigeninidin, and caffeic acid (Hargrove et al., 2011; Punia et al., 2020; Pontieri et al., 2021). While profiling could provide valuable insights into composition-activity relationships, we believe that using well documented sorghum phenolic extracts aligns more closely with the practical needs of the industry. Profiling has been very well documented by earlier studies, and our primary goal was to evaluate the extracts’ potential in a feedlot setting. In this study, our primary aim was to assess the overall phenolic profile and its associated biological activity, rather than to identify and quantify individual compounds. Therefore, we did not perform HPLC or LC-MS/MS analyses. By focusing on application driven outcomes, we aim to directly contribute to improving cattle health and feeding efficiency, rather than conducting a comprehensive chemical analysis. This approach ensures the research re-mains grounded in real-world applicability for the cattle industry.

The antioxidant activity of phenolic extracts from sorghum varies widely depending on genotype, flour, bran, and whole grain. Bran extracts, with higher phenolic content, show greater antioxidant activity than the flour. While the mechanism remains unclear, research highlights the antioxidant, anti-inflammatory, and anti-cancer effects of sorghum phenolic extracts, including phase II enzyme induction, p53 regulation, and cancer cell apoptosis (Jiang et al., 2020; Xu et al., 2021; Rezaee et al., 2024). This study confirms that specialty sorghum phenolic extracts possess notable antimicrobial effects against both subspecies of *F. necrophorum* (*necrophorum* and *funduliforme*), as well as *T. pyogenes*. These findings align with previous research demonstrating the antimicrobial properties of sorghum phenolics, particularly against anaerobic Gram-negative bacteria like *F. necrophorum*. Interestingly, our results showed no significant effect of sorghum phenolic compounds on the aerobic Gram-negative bacterium *Salmonella* Lubbock, which is consistent with earlier studies (Schnur et al., 2021). This suggests that the antibacterial activity of sorghum phenolics may vary based on the oxygen requirements and metabolic pathways of the bacteria. The differential susceptibility patterns observed between anaerobic (*Fusobacterium*) and aerobic (*Salmonella*) conditions could be attributed to physiological changes in the bacteria under these distinct incubation conditions (DeMars et al., 2016). Additional research supports the antimicrobial potential of sorghum phenolics. For example, sorghum phenolic extracts have been effective against *Enterococcus faecalis*, *Staphylococcus aureus*, *Campylobacter jejuni*, and *Campylobacter coli* in agar-well diffusion assays (Schnur et al., 2021). Furthermore, sorghum grain phenolic extracts inhibited *Clostridium perfringens* and *Salmonella enterica* in disc diffusion tests according to CLSI guidelines (Shields et al., 2020). In the meat industry, black and brown sumac sorghum bran have been shown to affect the physicochemical properties of beef sausage, enhancing texture attributes such as chewiness, hardness, resilience, cohesiveness, and gumminess, while also promoting oxidation, discoloration, and pH fluctuations. Microbiologically, sorghum bran inhibited *Listeria* spp. and *Escherichia coli* throughout storage and suppressed yeast, mold, and total coliform counts for 5–10 days (Xiang et al., 2022). Previous studies have also documented the antimicrobial properties of sorghum syrup against both Gram-negative and Gram-positive bacteria, further supporting the potential of sorghum-derived compounds as natural antimicrobial agents (Kumar et al., 2012). Additionally, sorghum’s glycine and proline rich protein components have been shown to exhibit antimicrobial activity against various bacteria, including *Bacillus subtilis*, *Rhodococcus fascians*, and *Escherichia coli* (Halder et al., 2019). Taken together, these findings suggest that the antimicrobial effects of sorghum phenolics are influenced by the bacterial species, their metabolic state, and the incubation conditions. The differential effectiveness against aerobic versus anaerobic bacteria highlights the need for further investigation into the specific mechanisms of action and the potential applications of sorghum phenolics in controlling pathogenic bacteria in diverse environments.

The activity of phenolic compounds and antioxidants in sorghum correlates with the thickness and color of the pericarp, with darker and thicker pericarps indicating higher levels of flavan-4-ols and anthocyanins, which provide greater health benefits (Dykes et al., 2005; Rao et al., 2018; Luo et al., 2022). Previous studies have demonstrated that dark colored grain sorghum is rich in antioxidants and phenolic compounds. For instance, sorghum bran color reflects differences in polyphenol composition among genotypes: brown sumac is high in proanthocyanidins, while black sorghum contains 3-deoxyanthocyanins such as luteolinidin and apigeninidin (Awika et al., 2003; Awika et al., 2005; Ghimire et al., 2021). The sorghum genotype and growing environment influence the availa-bility of these phenolic compounds, thereby affecting the grain’s appearance, color, and nutritional quality (Ghimire et al., 2021).

Our study concludes that sorghum phenolic extracts exhibit significant antimicrobial activity against liver abscess causing pathogens. The observed effects are likely attributable to the bioactive phenolic compounds present in the extracts. Although the exact mechanisms of action were not investigated in this study, existing literature suggests that phenolic compounds may exert their antimicrobial effects through multiple pathways. These include disrupting bacterial cell membranes, interfering with enzymatic activity, or inhibiting nucleic acid synthesis. The specific mechanism may vary depending on the unique structural and metabolic characteristics of each pathogen. Specifically, at a concentration of 1 mg/mL, extracts from black and brown sumac sorghum effectively inhibited both subspecies of *F. necrophorum* (*necrophorum* and *funduliforme*) and *Trueperella pyogenes*. In contrast, at a lower concentration of 0.1 mg/mL, the extracts only showed inhibition of *T. pyogenes* and subsp. *funduliforme*, but not the subspecies *necrophorum* or *Salmonella*. This variation may be due to factors such as pH, temperature, and storage time affecting the stability and degradation of the phenolic compounds. The differential susceptibility observed between *Fusobacterium necrophorum* and *Salmonella enterica* serotype Lubbock may be attributed in part to differences in their cell wall structures. As a Gram-negative, facultatively anaerobic bacterium, *Salmonella* possesses an outer membrane that can act as a barrier to many antimicrobial agents, including phenolic compounds. In contrast, *F. necrophorum* is an obligate anaerobe with distinct physiological characteristics and a less complex outer membrane structure, which may make it more susceptible to the phenolic extracts. Additionally, differences in metabolic pathways and oxygen requirements between these two organisms may also contribute to the observed variation in sensitivity.

Our findings highlight the potential of black and brown sumac sorghum extracts for controlling liver abscesses, demonstrating a dose dependent antibacterial effect. A limitation of this study is the lack of evaluation of the safety of sorghum phenolic extracts in terms of cell cytotoxicity, especially at the concentrations tested. While the focus was on their antimicrobial activity, understanding their safety profile is equally important for practical applications in livestock systems. Future research should include cytotoxicity assays using relevant cell lines to ensure the extracts are non-toxic and safe for use, which is a crucial step toward their adoption as sustainable alternatives to antibiotics. Additionally, exploring the antibacterial activity of different sorghum genotypes through *in-vivo* experiments could provide valuable insights for future studies and practical applications.

## 5 Conclusion

Liver abscesses in feedlot cattle are linked to high cereal grain diets, which can lower ruminal pH and damage ruminal epithelial tissue, facilitating bacterial entry into the bloodstream and subsequent liver abscess formation. While antibiotics like tylosin have been effective in controlling these abscesses, the rise of antibiotic resistance has fueled interest in alternative approaches to mitigate their negative impacts. Sorghum, a widely used feed grain, offers promising potential due to its rich content of phenolic compounds with antioxidant, anti-inflammatory, and antimicrobial properties. Our study demonstrates that phenolic extracts from specialty sorghum varieties, particularly black and brown sumac, exhibit significant antimicrobial activity against liver abscess causing pathogens, including both subspecies of *F. necrophorum* and *T. pyogenes*. The antimicrobial effectiveness of these extracts is dose dependent, with higher concentrations providing greater inhibition. These findings underscore the potential of sorghum phenolic extracts as natural antimicrobial agents and contribute to the growing body of research supporting the use of natural compounds as alternatives to antibiotics in livestock. This is crucial in the ongoing effort to mitigate antimicrobial resistance in animal agriculture. The study highlights the need for further research into the mechanisms of action and applications of these extracts, with exploration of different sorghum genotypes and plant parts offering valuable insights. Such investigations could lead to sustainable, effective solutions for managing livestock health and enhancing production efficiency, ultimately improving animal welfare and food safety.

## Data Availability

The original contributions presented in the study are included in the article/supplementary material. Further inquiries can be directed to the corresponding author.

## Author's note

Mention of trade names or commercial products in this publication is solely for the purpose of providing specific information and does not imply recommendation or endorsement by the U.S. Department of Agriculture. USDA is an equal opportunity provider and employer.
